# *Arabidopsis* cytosolic acyl-CoA-binding proteins ACBP4, ACBP5 and ACBP6 have overlapping but distinct roles in seed development

**DOI:** 10.1042/BSR20140139

**Published:** 2014-12-23

**Authors:** An-Shan Hsiao, Richard P. Haslam, Louise V. Michaelson, Pan Liao, Qin-Fang Chen, Sanjeewani Sooriyaarachchi, Sherry L. Mowbray, Johnathan A. Napier, Julian A. Tanner, Mee-Len Chye

**Affiliations:** *School of Biological Sciences, The University of Hong Kong, Pokfulam, Hong Kong, China; †Department of Biological Chemistry, Rothamsted Research, Harpenden, Hertfordshire AL5 2JQ, U.K.; ‡Department of Cell and Molecular Biology, Uppsala University, Box 596, Biomedical Center, 751 24 Uppsala, Sweden; §Science for Life Laboratory, Uppsala University, Box 596, Biomedical Center, 751 24 Uppsala, Sweden; ║Department of Biochemistry, The University of Hong Kong, Pokfulam, Hong Kong, China

**Keywords:** acyl-CoA-binding protein, isothermal titration calorimetry, lipid metabolism, seed, ABA, abscisic acid, ABRC, Arabidopsis Biological Resource Center, ACB, acyl-CoA-binding, ACBP, acyl-CoA-binding protein, CBF, C-repeat binding factor, CRT, C-repeat, EMSA, electrophoretic mobility shift assay, ER, endoplasmic reticulum, GUS, β-glucuronidase, ITC, isothermal titration calorimetry, MBS, MYB-binding site, PC, phosphatidylcholine

## Abstract

Eukaryotic cytosolic ACBPs (acyl-CoA-binding proteins) bind acyl-CoA esters and maintain a cytosolic acyl-CoA pool, but the thermodynamics of their protein–lipid interactions and physiological relevance in plants are not well understood. *Arabidopsis* has three cytosolic ACBPs which have been identified as AtACBP4, AtACBP5 and AtACBP6, and microarray data indicated that all of them are expressed in seeds; *AtACBP4* is expressed in early embryogenesis, whereas *AtACBP5* is expressed later. ITC (isothermal titration calorimetry) in combination with transgenic *Arabidopsis* lines were used to investigate the roles of these three ACBPs from *Arabidopsis thaliana*. The dissociation constants, stoichiometry and enthalpy change of AtACBP interactions with various acyl-CoA esters were determined using ITC. Strong binding of recombinant (r) AtACBP6 with long-chain acyl-CoA (C16- to C18-CoA) esters was observed with dissociation constants in the nanomolar range. However, the affinity of rAtACBP4 and rAtACBP5 to these acyl-CoA esters was much weaker (dissociation constants in the micromolar range), suggesting that they interact with acyl-CoA esters differently from rAtACBP6. When transgenic *Arabidopsis* expressing *AtACBP6pro::GUS* was generated, strong GUS (β-glucuronidase) expression in cotyledonary-staged embryos and seedlings prompted us to measure the acyl-CoA contents of the *acbp6* mutant. This mutant accumulated higher levels of C18:1-CoA and C18:1- and C18:2-CoAs in cotyledonary-staged embryos and seedlings, respectively, in comparison with the wild type. The *acbp4acbp5acbp6* mutant showed the lightest seed weight and highest sensitivity to abscisic acid during germination, suggesting their physiological functions in seeds.

## Abstract 2

The binding affinities of the three *Arabidopsis* cytosolic ACBPs (AtACBP4, AtACBP5 and AtACBP6) with acyl-CoA esters were investigated by ITC. When the biological significance of these AtACBPs was analysed using mutants, results indicated their overlapping functions in seed acyl-lipid metabolism.

## INTRODUCTION

ACBPs (acyl-CoA-binding proteins) have a highly conserved 10-kDa domain that binds long-chain acyl-CoA esters with high affinity [1–5]. ACBPs were first identified in mammals as a DBI (diazepam-binding inhibitor) or EP (endozepine) inhibiting diazepam binding to the GABA (γ-aminobutyric acid) receptor [6]. ACBPs were later demonstrated to be involved in multiple physiological processes such as fatty acid biosynthesis, enzyme regulation, intracellular acyl-CoA transport, and maintenance of intracellular acyl-CoA pools [7,8]. Homologues of ACBPs have been identified in eukaryotes (Animalia, Plantae, Fungi and Protista) as well as in Eubacteria, but not in Archaebacteria [7]. Multiple isoforms of ACBPs are present in fungi as well as in animals [9]. In mammals, three isoforms have been identified in different tissues: L-ACBP in liver, B-ACBP in brain and T-ACBP in testis [9]. The 10-kDa ACBP is a cytosolic protein that has been reported to be associated with the smooth ER (endoplasmic reticulum), Golgi bodies and the outer membrane of the mitochondria [10,11]. Bovine L-ACBP localizes to the ER and Golgi complex in a ligand-dependent manner [11].

Recombinant rat, bovine, human and *Plasmodium falciparum* ACBPs have been reported to bind to acyl-CoA esters *in vitro* [1,12–14]. Similar observations have been reported for recombinant AtACBPs using Lipidex assays in the model plant *Arabidopsis* [15–20]. In yeast, ACBPs are required for membrane assembly and fatty acid chain elongation [21]. ACBP-null mice lacking the 10-kDa ACBP were embryo-lethal, suggesting that the protein is essential during early development [22]. Besides lipid biosynthesis, membrane biogenesis, and the regulation of gene expression and enzyme activities, ACBPs also function in development and stress responses in *Caenorhabditis elegans* [23], mammals [24] and plants [25,26]. The appearance of ACBPs very early in evolution points to their fundamental role in acyl-CoA metabolism [7].

In *Arabidopsis*, six isoforms have been identified, designated as AtACBP1 to AtACBP6. Despite being conserved within the ACB (acyl-CoA-binding) domain, their molecular weights range from 10.4 to 73.1 kDa because of the additional domains in AtACBP1 to AtACBP5 [25]. The 37.5 kDa AtACBP1 and 38.5 kDa AtACBP2 share 72% amino acid identity throughout their sequences, and each includes a C-terminal domain of ankyrin repeats which mediates protein–protein interactions [15,18,27,28], and an N-terminal hydrophobic transmembrane domain that targets it to the ER and the plasma membrane [27,29–31]. Both proteins have been shown to participate in embryogenesis [30,32] and heavy metal stress tolerance [18,28,33], whereas AtACBP1 also plays a role in stem cuticle formation [34] and AtACBP2 in drought tolerance [35]. Their recombinant proteins have been reported to bind acyl-CoA esters *in vitro* as well as to PC (phosphatidylcholine) [15,18,29,32], while recombinant (r) AtACBP1 also binds to phosphatidic acid, and rAtACBP2 to lysophophatidylcholine (lysoPC) [28,32,36]. The 39.3-kDa AtACBP3 contains a cleavable N-terminal signal peptide and is the only member with an ACB domain localized at the C-terminus [17]. The extracellularly-targeted AtACBP3 [17,20,37] has been shown to bind acyl-CoA esters, PC and PE (phosphatidylethanolamine) [17,20].

The two largest AtACBPs, the 73.1-kDa AtACBP4 and 71.0-kDa AtACBP5, have an N-terminal ACB domain followed by two kelch motifs [16]. Both are localized in the cytosol [38]. Likewise, the 10-kDa AtACBP6 is cytosolic; this protein comprises only the ACB domain, and has well-characterized homologues in other species [39]. The recombinant forms of these three cytosolic proteins (AtACBP4, AtACBP5 and AtACBP6) [38,39] have been previously reported to bind acyl-CoA esters and PC in Lipidex assays [16,19,39]. Microarray data from the *Arabidopsis* eFP browser indicated that all three cytosolic *AtACBPs* are expressed in *Arabidopsis* seeds (http://bar.utoronto.ca/efp/cgi-bin/efpWeb.cgi).

Herein, the binding affinities of the three recombinant cytosolic AtACBPs to acyl-CoA esters were investigated by ITC (isothermal titration calorimetry), which represents a more precise method to measure protein–ligand binding compared with Lipidex assays [40]. To correlate the ITC data with the physiological roles of AtACBP6, its promoter was cloned and transgenic *Arabidopsis AtACBP6pro::GUS* lines were generated, allowing us to study the spatial pattern of expression. The acyl-CoA contents of the *acbp6* mutant were also measured in comparison with the wild-type. To investigate the biological significance of recombinant AtACBP4, AtACBP5 and AtACBP6 in the binding of various acyl-CoA esters, double and triple mutants of *AtACBP4*, *AtACBP5* and *AtACBP6* [41] were further analysed. Given indications of the overlapping functions of *AtACBP4*, *AtACBP5* and *AtACBP6* in flower development [41], the seed morphology and seedling development of these mutants were also studied.

## EXPERIMENTAL

### Preparation of recombinant (His)_6_-AtACBP4, (His)_6_-AtACBP5 and (His)_6_-AtACBP6 proteins

The protein expression plasmids containing full-length *AtACBP4* and *AtACBP5* cDNA were previously constructed in the vector pRSETB (Invitrogen) as pAT184 and pAT185, respectively [16]. Recombinant (His)_6_-AtACBP4 and (His)_6_-AtACBP5 proteins (rAtACBP4 and rAtACBP5) were expressed in *Escherichia coli*, purified and refolded according to Leung et al. [16]. The coding sequence of *AtACBP6* was amplified by PCR with primer pairs ACBP-F1 and ACBP-R1 and cloned into expression vector pEXP5-NT/TOPO (Invitrogen) to generate plasmid pEXP-*ACBP6*. Sequences of primers are listed in Supplementary Table S1. Recombinant (His)_6_-AtACBP6 (rAtACBP6) was expressed in *E. coli* and purified following Chye [29]. The three recombinant proteins were dialysed in 10 mM sodium phosphate buffer (pH 7.0). Protein concentrations were determined by the standard Bradford assay (Bio-Rad).

### ITC

Calorimetric titration was performed with a MicroCal iTC_200_ system (GE Healthcare) at 30°C. Long-chain acyl-CoA esters (Avanti Polar Lipids, Inc.) were dissolved in 10 mM sodium phosphate buffer (pH 7.0) to a final concentration of 0.25, 0.5 and 1 mM. The solution was degassed and loaded into a syringe, while 15 μM rAtACBP6, 6 μM rAtACBP4 or 60 μM rAtACBP5, was placed into the sample cell. For all titrations, 1.8 μl ligand was injected into the sample cell at 150 s intervals with a stirring speed of 1000 rpm. The titrations were completed after 20 injections. All data were collected automatically, and non-specific heat effects were estimated and corrected after saturation following the instructions of the manufacturer. Raw data were integrated and analysed using the Origin v.7.0 software accompanying the calorimeter.

### Plant material and growth conditions

Seeds of wild-type *Arabidopsis* Col-0 were purchased from the ABRC (Arabidopsis Biological Resource Center). The double and triple mutants of *AtACBP4*, *AtACBP5* and *AtACBP6* were generated by crossing the respective single mutants [41]. To sterilize *Arabidopsis* seeds, a solution containing 20% bleach and 0.1% (v/v) Tween 20 was used. Seeds were subjected to 20 min vigorous shaking in this solution, washed six times in distilled water, and then spread on MS (Murashige and Skoog) medium (Sigma-Aldrich) containing 2% (w/v) sucrose, and 0, 0.2 or 0.4 μM ABA (abscisic acid). After 4°C stratification for 2 days, the plates were placed under 16 h-light/8 h-dark cycles (22°C) in a tissue culture room. The rates of seed germination were scored when radicle protrusion occurred.

### Promoter analysis

A 1.5-kb 5′-flanking region of *AtACBP6* was amplified by PCR with primer pair ML795 and ML814. Deletion derivatives of the 1.5-kb 5′-flanking region of *AtACBP6*, 1.0, 0.6 and 0.3 kb in size were further amplified by PCR with corresponding forward primers ML1166, ML1167 and ML1168, and the reverse primer ML814. All forward primers contained a *Bam*HI site, and the reverse primer, a *Sma*I site. Sequences of primers are listed in Supplementary Table S1. Fragments were purified and cloned into the pGEM-T EASY Vector system (Promega). Each *Bam*HI–*Sma*I fragment was subcloned into corresponding restriction sites on the binary vector pBI101.3 (Clontech) to generate the *AtACBP6pro::GUS* fusion plasmids (pAT452, pAT590, pAT591 and pAT592) that were used in *Agrobacterium tumefaciens* transformation of wild-type *Arabidopsis* Col-0 by the floral dip method.

For GUS (β-glucuronidase) histochemical staining, samples of seedlings and embryos were immersed in a GUS substrate solution (50 mM sodium phosphate buffer, pH 7.0, 0.2% Triton X-100, 10 mM EDTA, 2 mM potassium ferricyanide, 2 mM potassium ferrocyanide and 1 mg/ml 5-bromo-4-chloro-3-indolyl-β-D-glucuronide), for 30 min with vacuum infiltration, after which samples were incubated at 37°C for 4 h (for seedlings) or 12 h (for embryos). Following a series of ethanol washes, tissues were observed and images were captured with a Nikon 80i Microscope.

Plant nuclear protein extraction was performed at 4°C using 10 g of 7-day-old *Arabidopsis* seedlings following the instructions of the Plant Nuclei Isolation/Extraction Kit (Sigma). Biotin-labelled DNA probes were prepared as specified in the Pierce Biotin 3′ End DNA Labeling Kit (Thermo Scientific). Probe 1 (ML2044/ML2045), Probe 2 (ML2046/ML2047) and Probe 3 (ML2048/ML2049) corresponded to each of the three putative Dof-boxes (−490/−486, −421/−417 and −353/−349, respectively). Sequences of the probes used are listed in Table S1. EMSAs (electrophoretic mobility shift assays) were performed on 6% native PAGE, and protein–DNA interactions subsequently revealed using the Chemiluminescent Nucleic Acid Detection Module (Thermo Scientific).

### Acyl-CoA profiling

Samples for acyl-CoA profiling were collected from 5-day-old seedlings and cotyledonary-staged embryos of wild-type Col-0 and the *acbp6* mutant. Samples were extracted following Larson and Graham [42]. Seedling samples were analysed by reversed-phase high-performance liquid chromatography [32], and embryo samples were analysed by liquid chromatography-tandem mass spectrometry with multiple reaction monitoring, operated in positive ion mode, according to Haynes et al. [43].

## RESULTS

### Recombinant AtACBPs bind to long-chain acyl-CoA esters

Long-chain acyl-CoA esters (C16- to C18-CoA) are major intermediates in lipid metabolism in plants [44], and cytosolic AtACBPs are potential participants in acyl-CoA trafficking, besides maintaining an acyl-CoA pool in the cytosol [8,25]. Therefore the binding of the recombinant cytosolic AtACBP4, AtACBP5 and AtACBP6 to these acyl-CoA esters was studied using ITC, to understand how their biochemical properties may be correlated with their physiological functions.

**Figure 1:**
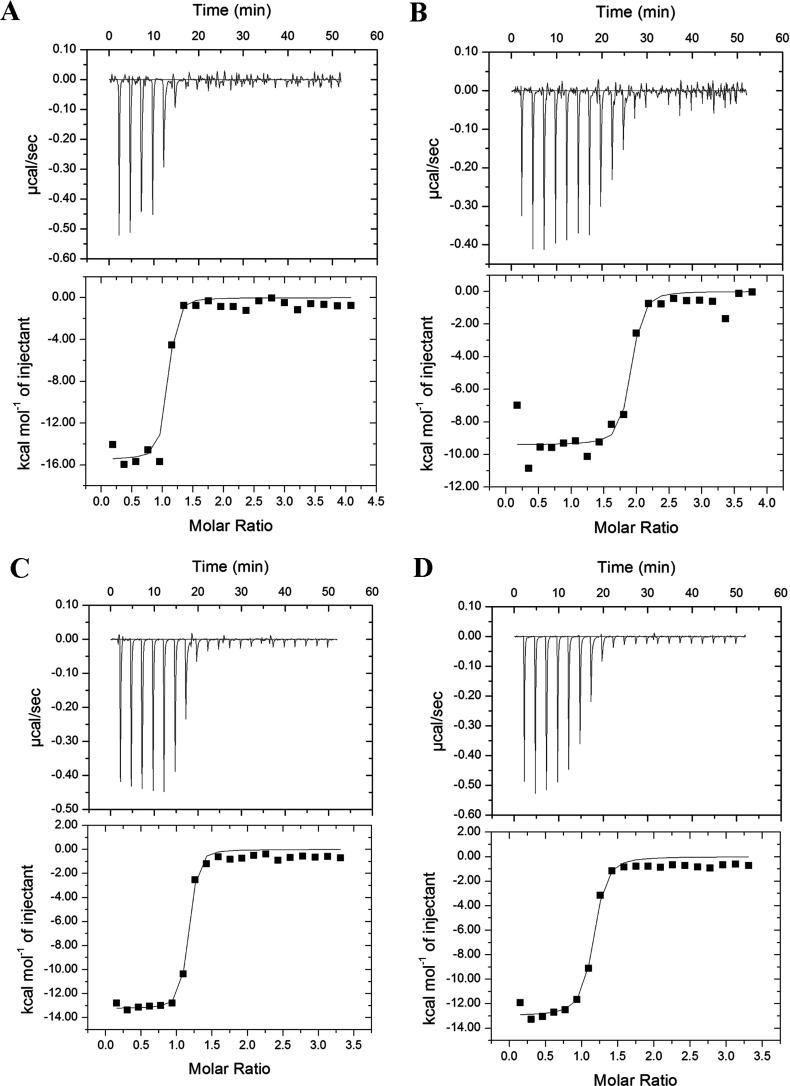
Binding isotherms of rAtACBP6 titrated with (A) palmitoyl-CoA (C16:0-CoA), (B) oleoyl-CoA (C18:1-CoA), (C) linoleoyl-CoA (C18:2-CoA) and (D) linolenoyl-CoA (C18:3-CoA) at 30°C Top panels show the raw data from 20 injections of 1.8-μl aliquots of a 0.25 mM (A, C and D) or 0.5 mM (B) solution of acyl-CoA into a cell containing 0.015 mM rAtACBP6 at 30°C; bottom panels show the integrated area of each injection after background correction.

ITC thermograms indicated that rAtACBP6 binds to palmitoyl-CoA (C16:0-CoA), oleoyl-CoA (C18:1-CoA), linoleoyl-CoA (C18:2-CoA) and linolenoyl-CoA (C18:3-CoA) (Figure 1). Theoretical fits to the experimental data were obtained using a single binding site model. Table 1 lists the thermodynamic parameters obtained, including dissociation constant (*K*_d_), stoichiometry of the ligand-to-protein binding (*n*), enthalpy change (Δ*H*), free energy change (Δ*G*) and the entropy change (Δ*S*). Our results indicated that rAtACBP6 has high binding affinity to long-chain acyl-CoA esters, with *K*_d_ values ranging from 35.9 to 84.1 nM (Table 1). Interestingly, among all the tested acyl-CoA esters, the interaction between rAtACBP6 and C18:1-CoA was unique, with *n*=1.9, suggesting that one molecule of rAtACBP6 binds to two acyl-CoA ligands (Table 1).

**Table 1:** Thermodynamic parameters for acyl-CoA binding to rAtACBP6 ITC experiments were carried out at 30°C in 10 mM sodium phosphate buffer (pH 7.0). The values are the means of three experiments±S.D.

Acyl-CoAs	*n* (kcal mol^−1^)	Δ*H* (cal mol^−1^ K^−1^)	Δ*S* (kcal mol^−1^)	Δ*G*	*K*_d_
14:0	1.0±0.0	−11.6±1.4	−4.6	−10.2±0.2	38.7±4.5
16:0	1.0±0.1	−13.7±2.4	−11.5	−10.2±0.0	35.9±8.4
18:0	1.0±0.0	−12.0±2.5	−6.6	−10.0±0.3	60.6±2.4
18:1	1.9±0.1	−6.7±3.8	11.4	−10.2±0.1	45.3±4.3
18:2	1.1±0.0	−12.3±1.4	−6.7	−10.2±0.2	36.4±4.9
18:3	1.1±0.0	−12.1±1.3	−7.7	−9.7±0.1	84.1±2.9

**Figure 2:**
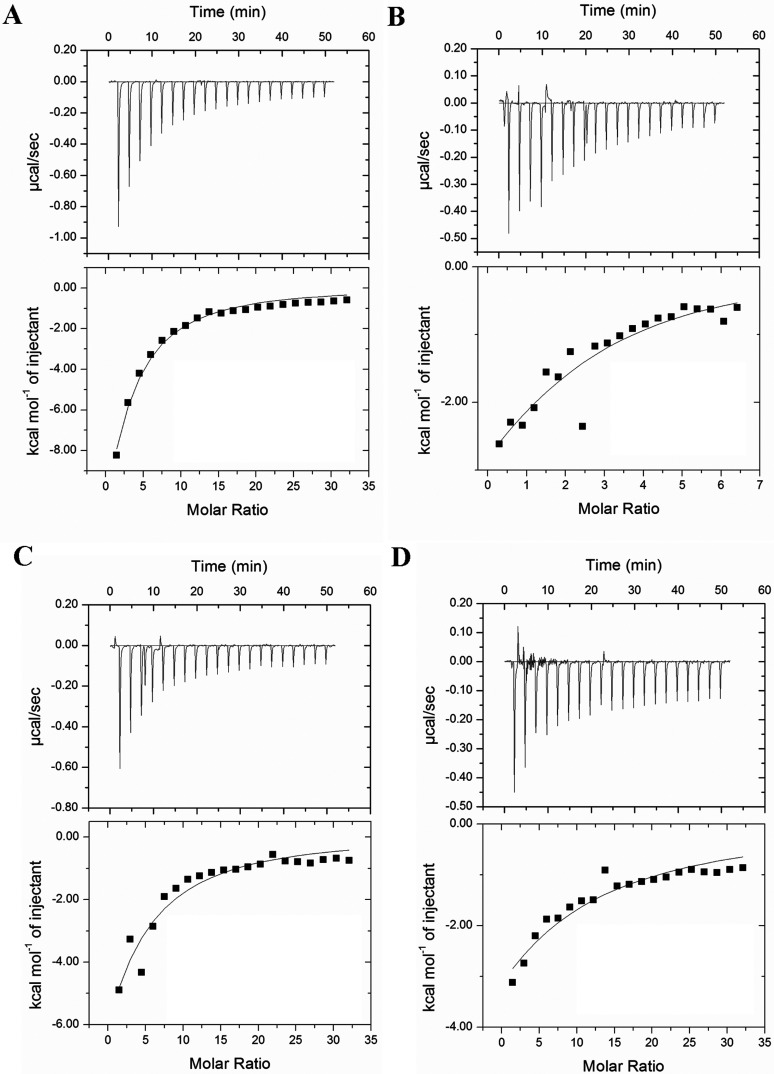
Binding isotherms of rAtACBP4 titrated with (A) palmitoyl-CoA (C16:0-CoA), (B) oleoyl-CoA (C18:1-CoA), (C) linoleoyl-CoA (C18:2-CoA) and (D) linolenoyl-CoA (C18:3-CoA) at 30°C Top panels show the raw data from 20 injections of 1.8-μl aliquots of a 1 mM solution of acyl-CoA into a cell containing 0.006 mM rAtACBP4 at 30°C; bottom panels show the integrated area of each injection after background correction.

**Figure 3:**
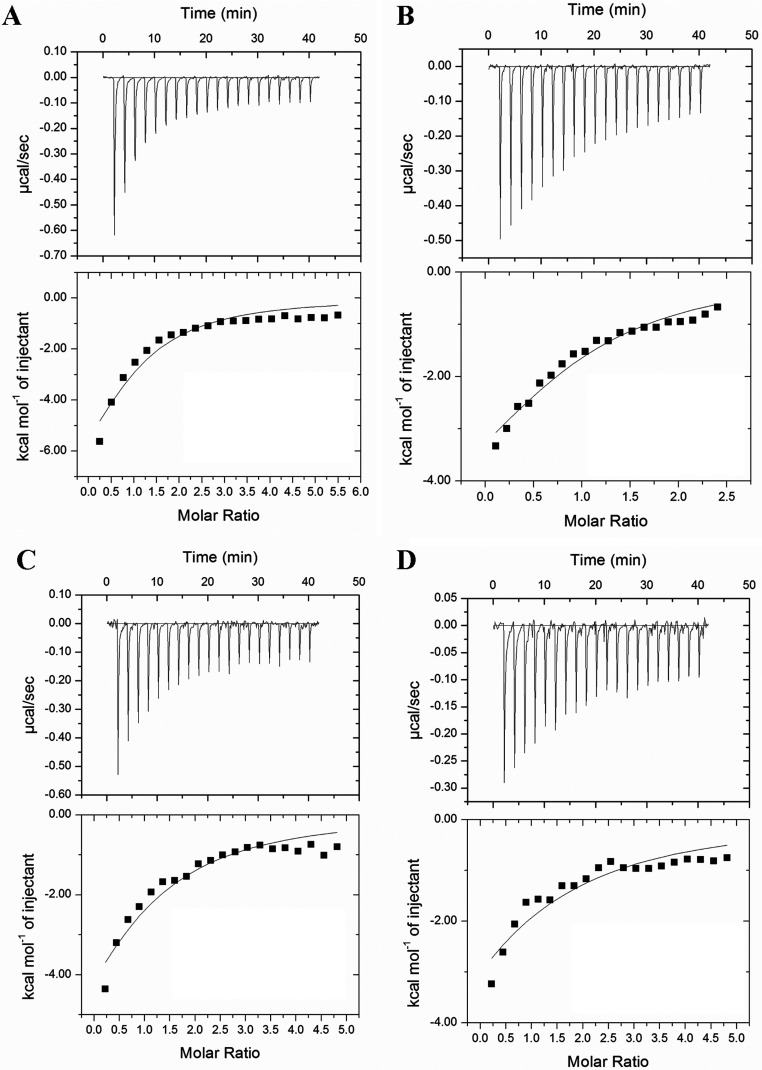
Binding isotherms of rAtACBP5 titrated with (A) palmitoyl-CoA (C16:0-CoA), (B) oleoyl-CoA (C18:1-CoA), (C) linoleoyl-CoA (C18:2-CoA) and (D) linolenoyl-CoA (C18:3-CoA) at 30°C Top panels show the raw heat signal from 20 injections of 1.8-μl aliquots of a 1 mM solution of acyl-CoA into a cell containing 0.06 mM rAtACBP5 at 30°C; bottom panels show the integrated area of each injection after background correction.

When the binding of rAtACBP4 and rAtACBP5 to acyl-CoA esters was investigated, ITC thermograms revealed that both bind to the same acyl-CoA esters tested for rAtACBP6 (Figures 2 and 3). Theoretical fits to the experimental data were again obtained using a single binding site model. Thermodynamic parameters for interaction between acyl-CoA esters and rAtACBP4 and rAtACBP5 are listed in Tables 2 and 3, respectively. The binding with both proteins was weaker than that observed for rAtACBP6. Recombinant AtACBP4 has affinities to long-chain acyl-CoA esters ranging from 2.7 to 189.0 μM (Table 2), while values for rAtACBP5 ranged from 35.4 to 92.4 μM (Table 3). An aberrant stoichiometry (*n*~2) for C18:1-CoA was observed for rAtACBP4, but not rAtACBP5.

**Table 2:** Thermodynamic parameters for acyl-CoA binding to rAtACBP4 ITC experiments were carried out at 30°C in 10 mM sodium phosphate buffer (pH 7.0). The values are the means of three experiments±S.D.

Acyl-CoAs	*n* (kcal mol^−1^)	Δ*H* (cal mol^−1^ K^−1^)	Δ*S* (kcal mol^−1^)	Δ*G*	*K*_d_
14:0	1.0±0.0	−53.5±6.9	−157.5	−5.8±0.1	65.3±22.6
16:0	1.0±0.0	−77.4±2.5	−235.5	−6.1±0.2	23.5±27.1
18:0	1.1±0.1	−8.9±1.0	−0.6	−8.7±1.0	2.7±1.6
18:1	2.1±0.0	−6.9±0.1	−4.3	−5.6±0.0	95.2±0.0
18:2	1.0±0.0	−73.7±2.6	−224.5	−5.8±0.2	73.8±0.3
18:3	1.0±0.0	−96.2±5.3	−300.5	−5.1±0.0	189.0±0.5

**Table 3:** Thermodynamic parameters for acyl-CoA binding to rAtACBP5 ITC experiments were carried out at 30°C in 10 mM sodium phosphate buffer (pH 7.0). The values are the means of three experiments±S.D

Acyl-CoAs	*n* (kcal mol^−1^)	Δ*H* (cal mol^−1^ K^−1^)	Δ*S* (kcal mol^−1^)	Δ*G*	*K*_d_
14:0	0.9±0.0	−3.7±1.2	7.7	−6.1±0.0	41.9±2.2
16:0	0.9±0.0	−11.2±0.5	−16.7	−6.2±0.0	35.7±1.0
18:0	1.0±0.0	−17.4±0.1	−37.2	−6.2±0.0	35.4±1.4
18:1	0.9±0.0	−6.2±0.2	−1.8	−5.7±0.0	74.4±3.5
18:2	1.0±0.0	−10.2±0.2	−14.7	−5.8±0.0	64.1±0.0
18:3	1.2±0.1	−7.8±1.3	−7.4	−5.6±0.1	92.4±10.7

### AtACBP6 was expressed in cotyledonary-staged embryos and seedlings

**Figure 4:**
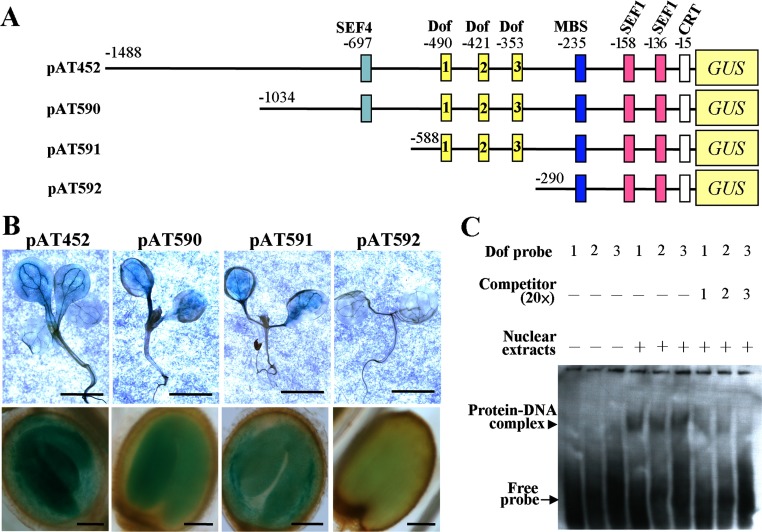
Analysis of the *AtACBP6* 5′-flanking region (**A**) Schematic diagram of *AtACBP6pro::GUS* constructs in which the *AtACBP6* 5′-flanking sequence was transcriptionally fused to the *GUS* reporter gene. Putative *cis-*elements on the *AtACBP6* 5′-flanking region (pAT452) and its deletion derivatives (pAT590, pAT591 and pAT592) are represented by boxes. SEF1, SOYBEAN EMBRYO FACTOR1; SEF4, SOYBEAN EMBRYO FACTOR4; Dof, DNA-binding with one finger; MBS, MYB-binding site; CRT, C-repeat; GUS, β-glucuronidase. The putative Dof-boxes are numbered 1, 2, and 3, corresponding to −490/−486, −421/−417 and −353/−349, respectively. (**B**) GUS staining of 7-day-old seedlings and cotyledonary-staged embryos from *T*_3_ generation of *Arabidopsis* transformants containing *AtACBP6pro::GUS* construct pAT452 and its deletion derivatives (pAT590, pAT591 and pAT592). Bar in the upper panel represents 5 mm. Bar in the bottom panel represents 100 μm. (**C**) EMSA analysis showing the binding of nuclear extracts to the Dof-boxes in the *AtACBP6* 5′-flanking region. Probe 1 (ML2044/2045), Probe 2 (ML2046/2047) and Probe 3 (ML2048/2049), containing the three Dof-boxes at −490/−486, −421/−417 and −353/−349, respectively, are numbered 1, 2 and 3. Competitor contains 20×non-biotin-labelled probe; −, no competitor in the reaction. Nuclear proteins were extracted from 7-day-old *Arabidopsis* seedlings; −, no nuclear proteins in the reaction; +, nuclear proteins added in the reaction. Arrowhead indicates the DNA–protein binding complex formed in the presence of nuclear proteins extracted from 7-day-old *Arabidopsis* seedlings. Arrow indicates free unbound biotin-labelled probe.

To correlate the ITC results on rAtACBP6 binding with its biological role, the spatial pattern of *AtACBP6* expression was elucidated using *AtACBP6pro::GUS* fusions in transgenic *Arabidopsis*. To this end, the 1.5-kb 5′-flanking region of *AtACBP6* and its three deletion derivatives were fused to *GUS* to generate plasmids pAT452, pAT590, pAT591 and pAT592 (Figure 4A). Analysis using the SoftBerry PlantProm database [45] suggested that the putative *cis*-elements that may be involved in *AtACBP6* regulation include the light-responsive DNA-binding with one finger boxes (Dof-boxes [46] at nucleotide positions −490/−486, −421/−417 and −353/−349 in Figure 4A), soya bean embryo factors SEF1 (−158/−150 and −136/−128) and SEF4 (−697/−686) which are motifs that bind plant embryo factors [47], and an MBS (MYB-binding site) (−235/−230), which binds the MYB transcription factor [48]. A putative CRT (C-repeat) element (−15/−11) associated with dehydration and freezing responses [49] was also identified (Figure 4A).

Histochemical staining of seedlings and the cotyledon-staged embryos from transgenic *Arabidopsis* expressing *AtACBP6pro::GUS* constructs derived from pAT452 and its deletion derivatives (pAT590 to pAT592) revealed that GUS was expressed in pAT452, pAT590 and pAT591 transformants, but not pAT592 transformants (Figure 4B), suggesting that loss of promoter activity occurred in the pAT592-transformed seedling and embryo. Also, pAT592 does not contain any putative Dof boxes, whereas pAT452, pAT590 and pAT591 contain all three Dof boxes; these Dof boxes may inherently affect the expression of *AtACBP6* during seed and seedling development.

### Dof-boxes are related to AtACBP6 expression

To investigate the function of the putative Dof-boxes in the *AtACBP6* 5′-flanking region, EMSAs were performed using double-stranded biotin-labelled DNA Probes 1, 2 and 3 representing each of the three Dof-boxes (−490/−486, −421/−417 and −353/−349, respectively). When crude nuclear extracts from 7-day-old seedlings were tested, band shifts caused by DNA–protein complexes were observed with all three probes (Figure 4C). Non-biotin-labelled probes outcompeted the binding of nuclear protein (Figure 4C), suggesting that the three Dof-boxes are involved in the expression of *AtACBP6*.

### C18:1-CoA ester accumulated in the *acbp6* seedlings and embryos

**Figure 5:**
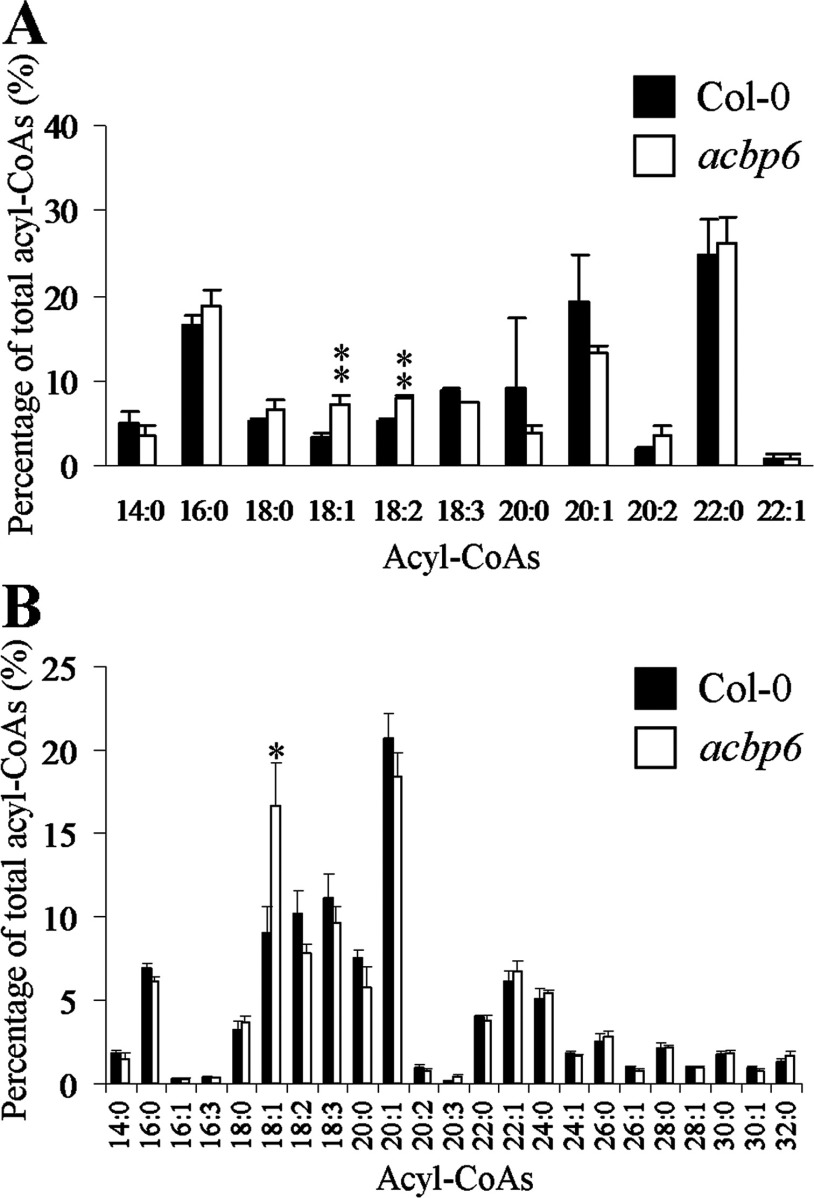
Acyl-CoA profiles of the *acbp6* mutant in comparison with wild-type *Arabidopsis* Col-0 (**A**) Acyl-CoA profiling in 5-day-old seedlings of the *acbp6* mutant in comparison to Col-0. Values are mean±S.D. of measurements made on three separate batches of samples. Student's *t* test for **, *P*<0.01. (**B**) Acyl-CoA profiling in cotyledonary-staged embryos of the *acbp6* mutant in comparison with Col-0. Values are mean±S.E.M. of measurements made on four separate batches of samples. Student's *t* test for *, *P*<0.05.

The pattern of GUS expression of *AtACBP6* in seedlings and the cotyledonary-staged embryos prompted us to test whether the acyl-CoA content was altered in 5-day-old seedlings and the cotyledonary-staged embryos of the *acbp6* mutant in comparison with the wild-type, using HPLC and liquid chromatography-tandem mass spectrometry. The results revealed that the *acbp6* mutant, but not the wild-type, accumulated C18:1-CoA in both 5-day-old seedlings and the cotyledonary-staged embryos (Figure 5). The *acbp6* mutant also showed C18:2-CoA accumulation in 5-day-old seedlings in comparison to the wild-type (Figure 5A). No other obvious differences were noted for other acyl-CoA esters in the cotyledonary-staged embryos (Figure 5B).

### The *acbp4acbp5acbp6* mutant showed the lightest seed weight

**Figure 6:**
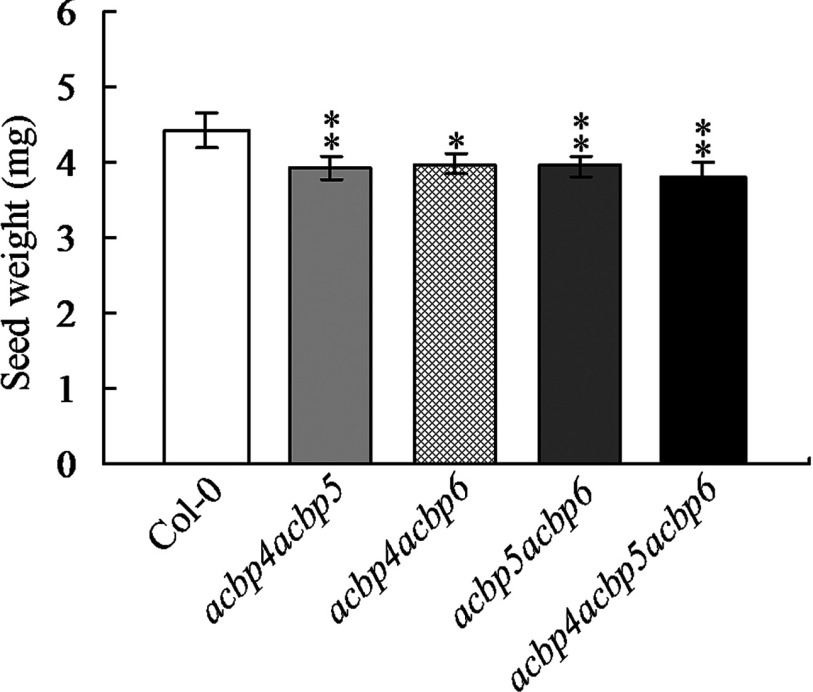
Seed weight of *acbp4acbp5*, *acbp4acbp6*, *acbp5acbp6* and *acbp4acbp5acbp6* mutants in comparison with wild-type *Arabidopsis* Col-0 Weight of 250 seeds from the *acbp4acbp5*, *acbp4acbp6*, *acbp5acbp6* and *acbp4acbp5acbp6* mutants versus Col-0. Data represent a mean value of four trials±S.D. Student's *t* test for *, *P*<0.05; **, *P*<0.01.

Results from microarray data on a comparison in the expression of *AtACBP4*, *AtACBP5* and *AtACBP6* in seeds (Supplementary Figure S1), together with observations that rAtACBP6, rAtACBP4 and rAtACBP5 bind to acyl-CoA esters, *AtACBP6pro::GUS* is highly expressed in cotyledonary-staged embryos, and acyl-lipid metabolism is important during seed development, prompted examination of the *acbp4acbp5*, *acbp4acbp6*, *acbp5acbp6* and *acbp4acbp5acbp6* mutants [41] to better understand the roles of these cytosolic AtACBPs in seed biology. There were no obvious variations in seed morphology and size among the double/triple mutants in comparison to the wild-type (Supplementary Figures S2 and S3). However, when seeds were weighed, the *acbp4acbp5acbp6* mutant seeds weighed less than wild-type (Figure 6).

### Hypersensitivity to ABA in seed germination in the double/triple mutants of AtACBP4, AtACBP5 and AtACBP6

**Figure 7:**
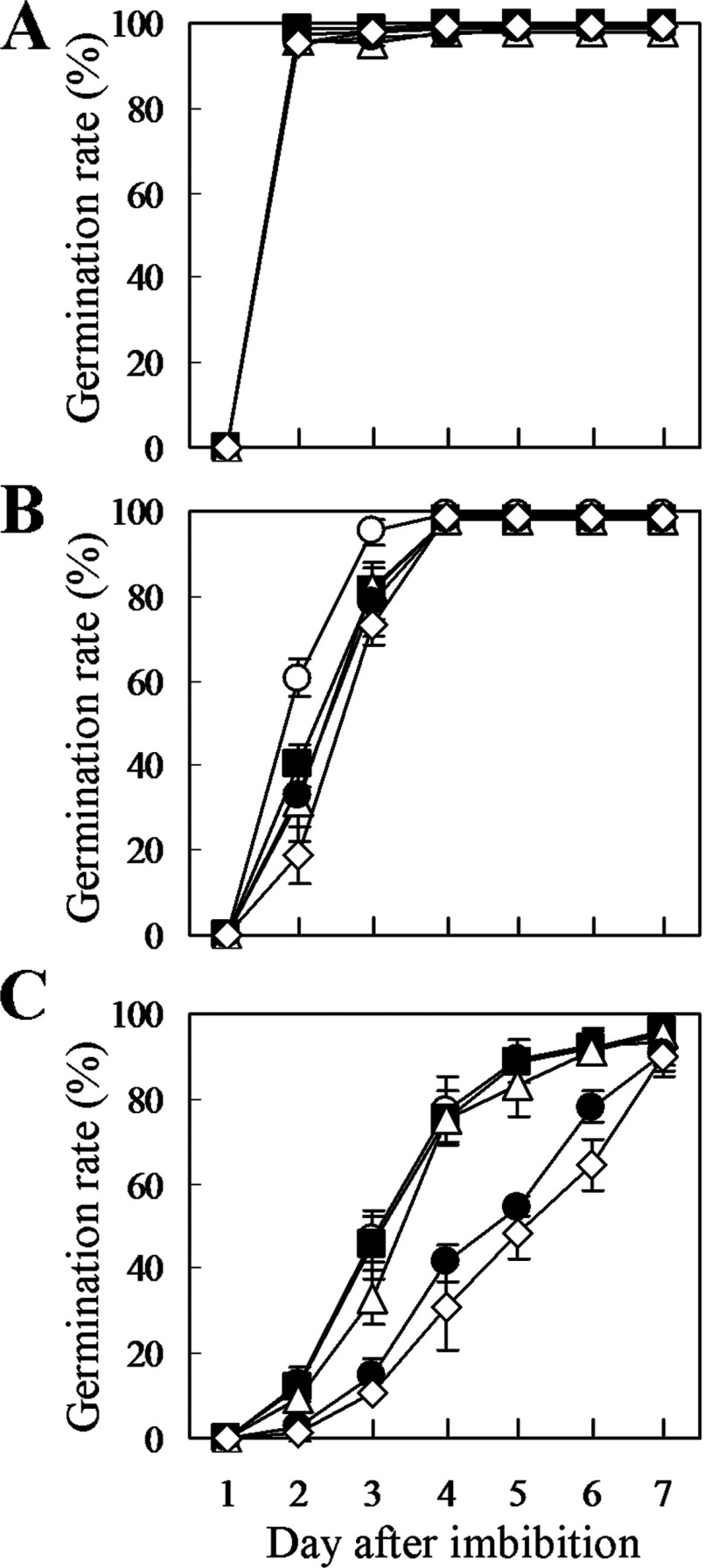
Germination rate of the double/triple mutants of cytosolic AtACBPs under ABA treatment in comparison with wild-type *Arabidopsis* Col-0 Seeds of Col-0 (open circle), the *acbp4acbp5* (closed square), *acbp4acbp6* (open triangle), *acbp5acbp6* (closed circle) and *acbp4acbp5acbp6* (open rhombus) mutants were cultured on MS medium supplemented with 0 (**A**), 0.2 (**B**) or 0.4 (**C**) μM ABA. Values are mean±S.D. of measurements of four separate batches of 33–44 seeds.

ABA treatment was used to evaluate the physiological roles of *AtACBP4*, *AtACBP5* and *AtACBP6* during seed germination. Under normal conditions, all double/triple mutants showed a similar rate in seed germination to Col-0 (Figure 7A). However, the germination of the mutants was delayed by ABA treatment. As shown in Figure 7(B), only 19% of *acbp4acbp5acbp6* seeds germinated within 2 days on the medium containing 0.2 μM ABA, compared with 60% of Col-0 seeds. Double mutants of *acbp4acbp5*, *acbp4acbp6* and *acbp5acbp6* showed intermediate germination rates of 40, 32 and 33%, respectively (Figure 7B). At a 0.4 μM ABA concentration, all the double and triple mutants showed lower germination rates in comparison with Col-0, whereas the triple mutant showed highest sensitivity to ABA (Figure 7C), suggesting that these three cytosolic AtACBPs play overlapping roles in seed germination.

## DISCUSSION

### Cytosolic AtACBPs bind to acyl-CoA esters *in vitro*

The small (~10 kDa), highly conserved cytosolic ACBPs from ox, rat, armadillo and yeast have been reported to bind long-chain acyl-CoA esters with high affinity using Lipidex assays [1,2], fluorescence and CD spectroscopy studies [3], as well as titration microcalorimetry [4,5]. In the present study, the *Arabidopsis* homologue, AtACBP6, was shown in ITC-binding assays to bind tightly to several long-chain acyl-CoA esters with *n*=1 (Figure 1; Table 1). However, for C18:1-CoA, the observation of *n*=1.9 (Table 1) suggests a binding stoichiometry of 2 moles of C18:1-CoA to 1 mole of rAtACBP6. That multiple molecules of ligand and protein can be involved in some cases is supported by earlier structural work with myristoyl-CoA and human cytosolic liver ACBP [50]. In the acyl-CoA profiling of the cotyledonary-staged embryos, C18:1-CoA was identified as the only acyl-CoA ester that accumulated in the *acbp6* mutant in comparison with the wild type (Figure 5B), suggesting that the physiological function of AtACBP6 in the cotyledonary-staged embryos is indeed related to this compound. In the acyl-CoA profiling of seedlings, both C18:1-CoA and C18:2-CoA accumulated in the *acbp6* mutant when compared with the wild type, suggesting the physiological function of AtACBP6 in seedlings is related to the binding of C18:2-CoA, as well as C18:1-CoA (Figure 5A). In plants, the cytosolic acyl-CoA pool includes C18:1-CoA derived from *de novo* fatty acid biosynthesis and acyl-editing, and C18:2-CoA, the product of acyl-editing [51]. The results of the acyl-CoA profiling and binding assay suggest that AtACBP6 plays a major role in maintaining the cytosolic acyl-CoA pool during seed and seedling development; this is supported by its strong expression in the embryo and seedling as demonstrated using *AtACBP6pro*::*GUS* transformants.

The recombinant 10-kDa ACBP from *Brassica napus* has been reported to bind acyl-CoA esters *in vitro* and enhance acyl exchange between acyl-CoA and PC through incorporation of C18:1-CoA [52]. Previous studies have indicated that AtACBP6 responds to freezing stress; furthermore AtACBP6 overexpressors exhibit a decline of PC in rosettes [39] and an elevation of PC in flowers [53] after freezing. Recombinant AtACBP6 has been reported to bind PC in filter-binding assays [39]. We demonstrate here by ITC that rAtACBP6 binds to acyl-CoA esters, suggesting that this protein may also function in maintaining a balance between acyl-CoA esters and PC.

Recombinant ACBPs, rAtACBP6 (Figure 1; Table 1) as well as rAtACBP4 (Figure 2; Table 2) and rAtACBP5 (Figure 3; Table 3) showed binding affinity to C16:0-CoA, C18:1-CoA, C18:2-CoA and C18:3-CoA. However, the binding trend of rAtACBP6 (C16:0~C18:2>C18:1>C18:3), rAtACBP4 (C16:0>C18:2>C18:1>C18:3) and rAtACBP5 (C16:0>C18:2>C18:1>C18:3) revealed in ITC analysis differed from our previous results of rAtACBP6 (C18:2>C18:1~C16:0>C18:3), rAtACBP4 (C18:1>C16:0>C18:2>C18:3) and rAtACBP5 (C18:1>C16:0>C18:2>C18:3) in Lipidex assays [16,19]. This variation may have arisen from the difference in the methods used. ITC can measure heat change directly during the binding interaction and therefore provides direct access to thermodynamic parameters and *K*_d_ [54], while the Lipidex assay is more likely to have imprecise *K*_d_ values since it is based on competition and not directly measuring the binding event [25,54].

The binding affinity of the 10-kDa bovine ACBP for C16:0-CoA was estimated to be 4.5 pM [5], while armadillo ACBP showed a range between 34 and 75 nM [3], and yeast ACBP gave 5.5 nM [4]. In *Arabidopsis*, the binding affinity of the 10-kDa rAtACBP6 was estimated by ITC to be 35.9 nM (Table 1), similar to armadillo ACBP [3]. In contrast, the binding affinity of rAtACBP4 to C16:0-CoA was 23.5 μM (Table 2) and that of rAtACBP5, 35.7 μM (Table 3), which represents 1000-fold weaker binding than armadillo ACBP. The substantially larger molecular weights of AtACBP4 (73 kDa) and AtACBP5 (71 kDa) reflect the presence of multiple structural/functional domains, and both of these recombinant proteins had to be purified from refolded inclusion bodies [16], in contrast to the soluble fraction for rAtACBP6. It is possible that this fact explains the relatively weaker binding for rAtACBP4 and rAtACBP5. However, the stronger binding affinity of rAtACBP6 to long-chain acyl-CoA esters compared to rAtACBP4 and rAtACBP5 may suggest that AtACBP6 interacts with acyl-CoA esters differently from AtACBP4 and AtACBP5. As only AtACBP6 responds to freezing stress [39,53], the stronger binding affinity of rAtACBP6 to long-chain acyl-CoA esters may imply that tight acyl-lipid binding becomes more critical under stress conditions than AtACBP4 and AtACBP5.

### The Dof-boxes in the *AtACBP6* 5′-flanking region bind nuclear proteins

The expression of *AtACBP6* has been reported based on Northern blot analysis to be cold inducible [39] and AtACBP6-overexpressing rosettes [39] and flowers [53] have been shown to be conferred freezing tolerance. When AtACBP6-overexpressing flowers and rosettes were subject to freezing treatment, the expression of one or more cold-related CBFs (C-repeat binding factors) [49,55] appeared to be affected [53]. In AtACBP6-overexpressing flowers, the expression of *CBF1*, *CBF2* and *CBF3* was higher than the wild-type after cold acclimation and freezing, while in AtACBP6-overexpressing rosettes, only *CBF3* showed higher expression than the wild-type after freezing and recovery [53]. CBF3 is known to bind the CRT element and CBF-induced genes contain one or more putative CRT elements in their 5′-flanking regions [55]. Using the PlantProm database, a putative CRT element (−15/−11) was predicted in the 5′-flanking region of *AtACBP6*, suggesting that this CRT may be involved in the *AtACBP6*-mediated freezing response.

Furthermore, analysis of the 5′-flanking region of *AtACBP6* revealed that the Dof-boxes function in regulating *AtACBP6* expression (Figure 4). A previous report has demonstrated that the Dof-box activates dark-responsive regulation of *AtACBP3* expression [56], suggesting the importance of Dof-boxes in AtACBP regulation. Dof proteins are unique to plants [57], and members form a major family of transcription factors [46]. Their diverse roles in plants include light responses, phytohormone and defence responses, and seed development and germination [46,58]. Although only one Dof-box (−341/−338) was shown to regulate *AtACBP3* expression in response to darkness [56], all three Dof-boxes (−490/−486, −21/−417 and −353/−349) in the *AtACBP6* 5′-flanking region were shown here using EMSAs to bind nuclear proteins from seedlings (Figure 4). In addition, the loss of the Dof-boxes at −490/−486, −421/−417 and −353/−349 adversely affected *AtACBP6pro::GUS* expression in 7-day-old seedlings and embryos (Figure 4).

### Acyl-CoA transport during seed development to seed germination

It has been reported that recycled acyl groups (C16:0, C18:1, C18:2 and C18:3) in the acyl-CoA pool in the soybean embryo provide the majority of acyl chains for *de novo* glycerol-3-phosphate acylation, representing the major flux in TAG formation in oil bodies [59]. In *Arabidopsis*, AtACBP1 and AtACBP2 are expressed in the embryo [30,32], whereas *AtACBP4*, *AtACBP5* and *AtACBP6* are predicted (Supplementary Figure S1) to be expressed during seed development. To address the role of the three cytosolic AtACBPs in seeds, recombinant cytosolic AtACBPs (rAtACBP6, rAtACBP4 and rAtACBP5) were verified to bind long-chain acyl-CoA esters, i.e. C16:0-CoA, C18:1-CoA, C18:2-CoA, C18:3-CoA (Figures 1–3; Tables 1–3), suggesting that these AtACBPs can assist in the maintenance of an acyl-CoA pool and represent potential candidates for acyl-CoA trafficking during lipid metabolism from seed development to seed germination [25]. It was observed that rAtACBP6 exhibited binding to long-chain acyl-CoA (C16- to C18-CoA) esters with dissociation constants in the nanomolar range, the affinity of rAtACBP4 and rAtACBP5 to these acyl-CoA esters was much weaker (dissociation constants in the micromolar range), suggesting that they interact with acyl-CoA esters differently from rAtACBP6. Furthermore, microarray data indicated a difference in the expression pattern between *AtACBP4* and *AtACBP5* during embryogenesis; *AtACBP4* was expressed in early embryogenesis while *AtACBP5* was expressed in late embryogenesis (Supplementary Figure S1).

AtACBP6 may play an important role in acyl-CoA transport in seed and seedling development, given that C18:1-CoA was observed to accumulate in the *acbp6* embryos, while both C18:1-CoA and C18:2-CoA accumulated in the *acbp6* seedlings (Figure 5). Consistently, a decline in C18:1-CoA was observed in transgenic *Arabidopsis* developing seeds (20 days after flowering) that were ectopically expressing the *Brassica* 10-kDa ACBP [60]. Taken together, these results support a role for the 10-kDa ACBP in acyl-CoA transport in seeds. In seed germination assays under ABA treatment, all the double and triple mutants of cytosolic AtACBPs showed a more sensitive germination phenotype in comparison with the wild type (Figure 7), suggesting that the change in the acyl-CoA pool size may have contributed to their sensitivity to ABA during seed germination. The degree of sensitivity in the double/triple mutants was *acbp4acbp5acbp6*> *acbp5acbp6> acbp4acbp6*> *acbp4acbp5* (Figure 7), indicating that *AtACBP6* plays the most important role among the three cytosolic *AtACBPs*, and that *AtACBP5* may be more important than *AtACBP4*. Hence, it was not surprising that the *acbp4acbp5acbp6* mutant yielded the lowest seed weight (Figure 6). Taken together this study suggests that the three cytosolic AtACBPs have overlapping roles in seed acyl-lipid metabolism, which is intimately linked to their ability to bind acyl-CoA esters.
