# *Arabidopsis* cytosolic acyl-CoA-binding proteins ACBP4, ACBP5 and ACBP6 have overlapping but distinct roles in seed development

**DOI:** 10.1042/BSR035e00176

**Published:** 2015-02-25

**Authors:** A. S. Hsiao, R. P. Haslam, L. V. Michaelson, P. Liao, Q. F. Chen, S. Sooriyaarachchi, S. L. Mowbray, J. A. Napier, J. A. Tanner, M. L. Chye

Volume 34, issue 6 (2014) e00165

The units were omitted for Δ*G* and *K*_d_ in Tables 1–3. The correct units as follows:

Table 1: Δ*G* (kcal mol^−1^) and *K*_d_ (nM)

Table 2: Δ*G* (kcal mol^−1^) and *K*_d_ (μM)

Table 3: Δ*G* (kcal mol^−1^) and *K*_d_ (μM).

